# TRPA1 and TRPV1 contribute to propofol-mediated antagonism of U46619-induced constriction in murine coronary arteries

**DOI:** 10.1371/journal.pone.0180106

**Published:** 2017-06-23

**Authors:** Pritam Sinharoy, Ian N. Bratz, Sayantani Sinha, Loral E. Showalter, Spencer R. Andrei, Derek S. Damron

**Affiliations:** 1 Department of Anesthesia, Perioperative and Pain Medicine, Stanford School of Medicine, Stanford, California, United States of America; 2 Department of Integrative Medical Sciences, Northeast Ohio Medical College, Rootstown, Ohio, United States of America; 3 Department of Surgery, Division of Orthopedic Surgery, Children’s Hospital of Philadelphia, Philadelphia, Pennsylvania, United States of America; 4 Department of Biological Sciences, Kent State University, Kent, Ohio, United States of America; Indiana University School of Medicine, UNITED STATES

## Abstract

**Background:**

Transient receptor potential (TRP) ion channels have emerged as key components contributing to vasoreactivity. Propofol, an anesthetic is associated with adverse side effects including hypotension and acute pain upon infusion. Our objective was to determine the extent to which TRPA1 and/or TRPV1 ion channels are involved in mediating propofol-induced vasorelaxation of mouse coronary arterioles *in vitro* and elucidate the potential cellular signal transduction pathway by which this occurs.

**Methods:**

Hearts were excised from anesthetized mice and coronary arterioles were dissected from control C57Bl/6J, TRPA1^-/-^, TRPV1^-/-^ and double-knockout mice (TRPAV^-/-^). Isolated microvessels were cannulated and secured in a temperature-controlled chamber and allowed to equilibrate for 1 hr. Vasoreactivity studies were performed in microvessels pre-constricted with U46619 to assess the dose-dependent relaxation effects of propofol on coronary microvascular tone.

**Results:**

Propofol-induced relaxation was unaffected in vessels obtained from TRPV1^-/-^ mice, markedly attenuated in pre-constricted vessels obtained from TRPA1^-/-^ mice and abolished in vessels obtained from TRPAV^-/-^ mice. Furthermore, NOS inhibition with L-NAME or endothelium denuding abolished the proporfol-induced depressor response in pre-constricted vessels obtained from all mice. In the absence of L-NAME, BK_Ca_ inhibition with penitrem A markedly attenuated propofol-mediated relaxation in vessels obtained from wild-type mice and to a lesser extent in vessels obtained from TRPV1^-/-^, mice with no effect in vessels obtained from TRPA1^-/-^ or TRPAV^-/-^ mice.

**Conclusions:**

TRPA1 and TRPV1 appear to contribute to the propofol-mediated antagonism of U46619-induced constriction in murine coronary microvessels that involves activation of NOS and BK_Ca_.

## Introduction

Transient receptor potential (TRP) ion channels have recently emerged as key regulators of vasomotor tone [1–6] and many general anesthetic agents modulate vasomotor tone [7–10]. Moreover, our laboratory and others have demonstrated that anesthetic agents activate and/or modulate TRPA1 and/or TRPV1 ion channel sensitivity to agonist activation in sensory neurons and heterologous expression systems [11–16]. However, a link between anesthetic agents and TRPA1 or TRPV1 activation in the modulation of vasomotor tone has yet to be established and virtually nothing is known about molecular interactions of anesthetics with TRP ion channels in the vasculature.

Propofol, an intravenous anesthetic, is extensively used during general anesthesia and outpatient procedures. The advantages of propofol sedation include rapid onset of action, improved patient comfort and rapid clearance, prompt recovery and cardioprotection by reducing oxidative stress due to its antioxidant properties [17]. However, unwanted side effects associated with the use of propofol include vasodilation resulting in hypotension, apnea and acute pain upon infusion [18]. The hypotensive response is of particular concern, particularly in patients with limited cardiovascular reserve and/or hemodynamic instability. At present, we have very little mechanistic evidence and understanding of the cellular signaling pathway(s) and molecular mechanism(s) by which propofol causes myocardial depression, hypotension and regulate coronary blood flow. Previously, we illustrated a role for TRPA1 ion channels in propofol-mediated depressor responses *in vivo* [1], but systematic studies performed *in vitro* specifically addressing the role of TRP ion channels as a target of anesthetic agents in the modulation of microcirculation have not been performed. Although, numerous studies have demonstrated that propofol-mediated activation of TRPA1 in sensory neurons and heterologous expression systems [11, 14], no studies have addressed the extent to which propofol modulate vasomotor tone in microvascular beds via direct interactions with TRP ion channels. Moreover, no studies have addressed the potential for cross-talk between TRPA1 and TRPV1 channels in mediating anesthesia-induced (propofol) hypotension.

In the current *in vitro* study, our goal was to examine the extent to which TRPA1 and/or TRPV1 ion channels mediate propofol-induced vasorelaxation of murine isolated coronary arterioles and to elucidate the intracellular signaling pathway(s) involved. The major finding is that propofol-induced relaxation was maintained in pre-constricted vessels obtained from TRPV1^-/-^ mice, markedly attenuated in those obtained from TRPA1^-/-^ mice and virtually abolished in the pre-constricted vessels taken from TRPAV^-/-^ (TRPA1/TRPV1) double knockout mice. Moreover, eNOS inhibition or denudation completely blocked the propofol-induced relaxation in all mice. The combination of NOS and BK_Ca_ inhibition prevented the propofol-induced relaxation in endothelium intact microvessels obtained from control mice. In the presence of combined BK_Ca_ channel and eNOS inhibition, the relaxation observed in pre-constricted vessels from TRPA1^-/-^ mice were virtually identical to those observed in microvessels obtained from control mice and were completely absent in microvessels obtained from the TRPAV^-/-^ double knockout mice. Altogether, this *in vitro* study suggests a complex interaction between TRPA1 and TRPV1 in mediating propofol-induced relaxation of murine coronary arterioles, and indicates a prominent involvement of eNOS and BK_Ca_ channels.

## Methods

### Mice

The Institutional Animal Care and Use Committee at Kent State University in accordance with The National Institutes of Health Guidelines approved all experiments and protocols for the Care and Use of Laboratory Animals. Experiments were performed in 8–12 week old males of C57Bl6, TRPA1^-/-^, TRPV1^-/-^ mice and in the double knockout (TRPA1/TRPV1) TRPAV^-/-^ mice [1].

### Coronary vessel pressure myography

Mice received inhalational anesthesia via 1.5–2.5% sevoflurane gas with supplemental oxygen and once anesthetized, were maintained under sevoflurane via nose cone. Hearts were excised from anesthetized mice, placed in ice-cold physiological salt solution and coronary arterioles were dissected free from ventricular wall tissue in buffer containing the following (in mM): 145 NaCl, 5.0 KCl, 2.5 CaCl_2_, 1.17 MgSO_4_, 25.0 NaHCO_3_, and 10 glucose, (pH 7.4). Isolated microvessels were cannulated and secured in a temperature-controlled chamber (Danish Myotech, DMT, Atlanta, GA) mounted on the stage of an inverted microscope outfitted with a video camera and edge detection analyzing software. Coronary arterioles were pressurized to 60 mmHg, warmed to 37°C and allowed to equilibrate for 1 hr. Vessel viability was examined using 60 mM KCl to induce contraction. Vasoreactivity studies were performed in endothelium intact and denuded coronary microvessels. Disruption of the endothelial layer was achieved by passing 1ml of air through the lumen. Denuded vessels were assessed by the addition of acetylcholine (1μM) to U46619 (1μM)-constricted arterioles.

### Mouse Coronary Artery Endothelial Cells (MCAECs)

MCAECs and endothelial growth cell medium were purchased from Cell Biologics Inc. (Campbell Park Drive, Chicago, IL) and were cultured according to the manufacturer’s instructions.

### Mouse aortic endothelial cells

Mouse aortic endothelial cells were isolated as previously described [19] with modifications. The aorta was isolated, placed in ice-cold HEPES solution, cleaned immediately and pooled in 1 ml of dissociation solution containing 1 mg/ml papain and 1 mg/ml dithiothreitol (30 mins, 37°C) then transferred into dissociation solution containing 1.5 mg/ml collagenase and 1 mg/ml soybean trypsin inhibitor and further incubated for 15 mins at 37°C. Afterwards, this solution was replaced with 2 ml of ice-cold HEPES solution and incubated (23°C, 10 min). The supernatant was then replaced with 1 ml HEPES solution, before trituration of vessel segments to release the cells. The cells were centrifuged at 500g for 5 mins and re-suspended in 60μl of endothelial cell media (DMEM Low glucose, 1% Antibiotic-Antimycotic, 10% Nu-Serum, 10% FBS, endothelial cell growth serum and heparin), centrifuged and again re-suspended in media. The cell suspension was incubated with magnetic beads containing the endothelial maker CD31 for 1 hr and then separated using an LS Column and MidiMacs Separator (CD31-Microbead Endothelial Isolation kit—Miltenyl Biotec, Auburn, CA). Cells were then seeded onto a 5 mm dish containing coverslips pre-coated with gelatin/fibronectin and verified via staining for the endothelial marker CD31.

### TRPA1 and TRPV1 antibodies

Synthetic peptides corresponding to Tyr455—Asp472 (YRPVEGLPPYKLNNTVGD) (V1-60) and Glu601—Ser627 (EDGKNNSLPVESPPHKCRGSACRPGNS) (V1-61) on the murine TRPV1 protein and Lys741 –Thr767 (KIQPGMAFNSTGIINGTSSTHEERIDT) (A1-62) and Gln898 –Ala924 (QDAFSTPLLSLIQTFSMMLGDINYRDA) (A1-63) on the murine TRPA1 protein were coupled to KLH and used to vaccinate rabbits to generate polyclonal antisera (Cleveland Clinic Hybridoma Core Facility, Cleveland, OH).

Peptides conjugated to KLH and corresponding to the same regions previously mentioned on the murine TRPV1 and TRPA1 proteins were obtained from the Cleveland Clinic and used to vaccinate C57Bl/6, TRPA1^-/-^, TRPV1^-/-^ and TRPAV^-/-^. Mice (3 per group) were vaccinated with 50 μg of peptide in a 100ul emulsion of Freund’s Complete Adjuvant placed subcutaneously in each flank. Every three weeks mice were similarly boosted with 25 μg of peptide in Incomplete Freunds Adjuvant. Blood samples were taken via submandibular bleeding 14 days after each boost. Mice were boosted a maximum of 4 times prior to sacrifice. Whole blood obtained from submandibular bleeds was allowed to coagulate for one hour at 41°C and then spun down at 2,000 X g for 10 minutes. Sera were removed, aliquoted, and stored at -70°C for future use. All subsequent experiments were performed with V1-60 and A1-62 antisera. Both rabbit and mouse primary antisera were used for immunoblot and immunocytochemical analysis.

### Immunoblot analysis

Immunoblot analysis was performed on whole cell lysates as mentioned previously [11]. Protein concentration was measured using the Bradford method and all sample concentrations were adjusted to 1–2 mg/ml in sample buffer. Equal amounts of protein (30 *μ*g) were separated by SDS-PAGE on 12% polyacrylamide gels and transferred to nitrocellulose membranes. Nonspecific binding was blocked with Tris-buffered saline solution (0.1% [vol/vol] Tween-20 in 20 mmol/L Tris base, 137 mmol/L NaCl adjusted to pH 7.6 with HCl, containing 5% [wt/vol] dry milk for 45 min. Membranes were incubated with primary antisera (Rabbit A1-62 and mouse V1-60) for 1 h followed by 3x10 min wash and incubation with horseradish-peroxidase-linked secondary antibody. Bound antibody was detected by enhanced chemiluminescence. All blots were analyzed using software (ImageJ; National Institutes of Health, Washington, DC).

### Immunocytochemistry of MCAECs

MCAECs were grown on coverslips for 24hrs and fixed with 4% paraformaldehyde for 45 min at 4°C, then blocked with phosphate-buffered saline containing 5% normal donkey serum and incubated overnight at 4°C with primary antibodies. Coverslips were washed both before and after incubation with fluorescently labeled secondary antibodies, mounted using vectashield and sealed before viewing under a confocal microscope. Images were acquired with fluorescence/confocal microscope (Olympus IX-70, FV5 PSU, Olympus America, Center Valley, PA) using appropriate excitation filters.

### Fluorescent activated cell sorting (FACS) analysis

Cultured MCAECs were fixed with 1:1 methanol and acetone for 10 minutes at 4°C then washed and blocked with 5% BSA in PBS. Cells were incubated with 20ul of antisera or corresponding isotype control antibody for 1 hr. at room temperature, washed, and then stained with anti-Rabbit IgG conjugated to FITC or anti-Mouse IgG conjugated to AlexaFluor-647. Detection of bound antisera was assessed by flow cytometry using an Amnis Flowsight running IDEAS software (EMD Millipore, Billerica, MA).

### Intracellular Ca^2+^ measurements in MCAEC’s

MCAECs were incubated in media (37°C, 5% CO_2_) for 45 min with fura-2 acetoxy methylester (fura-2/AM; 2 μM, TEFLabs, Austin, TX), washed and placed in a chamber (Warner Instruments, Hamden, CT) mounted on the stage of an Olympus IX-81 inverted fluorescence microscope (Olympus America, Center Valley, PA). The cells were superfused continuously with Hepes-buffered saline (HBSS) containing the following: 118 mM NaCl, 4.8 mM KCl, 1.2 mM MgCl_2_, 1.25 mM CaCl_2_, 11.0 mM dextrose, 5 mM pyruvate and 25 mM HEPES at a flow rate of 2 ml/min. The cells were exposed to respective channel agonists for 10 seconds, followed by a switch back to HBSS for washout. Intracellular free Ca^2+^ concentration ([Ca^2+^]_i_) was examined in individual endothelial cells using a fluorescence imaging system as previously described [11]. The fluorescence imaging system (Easy Ratio Pro, Photon Technology International, Lawrenceville, NJ, USA) equipped with a multi wavelength spectrofluorometer (DeltaRAM X) and a QuantEM 512SC electron multiplying CCD camera (Photometrics, Tuscon, AZ, USA). Representative photometric data were acquired by alternating excitation wavelengths between 340 and 380 nm (20 Hz) and monitoring an emission wavelength of 510 nm. The ratio of the light intensities at the two wavelengths was used to measure changes in [Ca^2+^]_i_. Prior to data acquisition, background fluorescence was measured and automatically subtracted from the subsequent experimental measurement using Easy Ratio Pro.

### Proximity ligation assay (PLA)

PLA was performed using Duolink reagents (Sigma-Aldrich) per manufacturer’s instruction. Cultured MCAECs were fixed with 4% paraformaldehyde at 4°C then washed and blocked with 5% BSA in PBS. Cells were incubated with rabbit (A1-62) and mouse (V1-60) primary antisera for overnight at 4°C. Coverslips were mounted using Vectashield and sealed before viewing under a confocal microscope Olympus IX-70, FV5 PSU, Olympus America, Center Valley, PA) using the filter set Cyanine 5 (650/670 nm).

### Drugs and chemicals

All drugs were purchased from Sigma Chemicals (St. Louis, MO) unless otherwise stated. Propofol stock solution (10 mg/ml, injectable emulsion) was purchased from Cleveland Clinic (Cleveland, OH).

### Data analysis and statistics

For the vasoreactive studies, an experimental group size of 12 animals was determined to be necessary to achieve at least 15% minimal difference in lumen diameter (α<0.05 and 90% power). The percent change in relaxation was calculated using the formula: % change = (Maximum-Base-line)/Base line times 100. All Ca^2+^ imaging experiments were repeated in a minimum of six separate coverslips of cells. Results obtained from each coverslip were averaged so that all coverslips of cells were weighted equally. Gaussian distribution was examined by the Shapiro—Wilk normality test. Data are expressed as means ± SEM. Statistical comparisons between the groups were made using repeated-measures two-way analysis of variance and the Bonferroni post hoc test. For statistical analyses, Sigma Plot 11.0 software (Systat Software, San Jose, CA) was utilized. A value of p < 0.05 was considered statistically significant.

## Results

### Propofol-mediated coronary vasoreactivity: Role of TRPA1 and TRPV1

**Fig 1:**
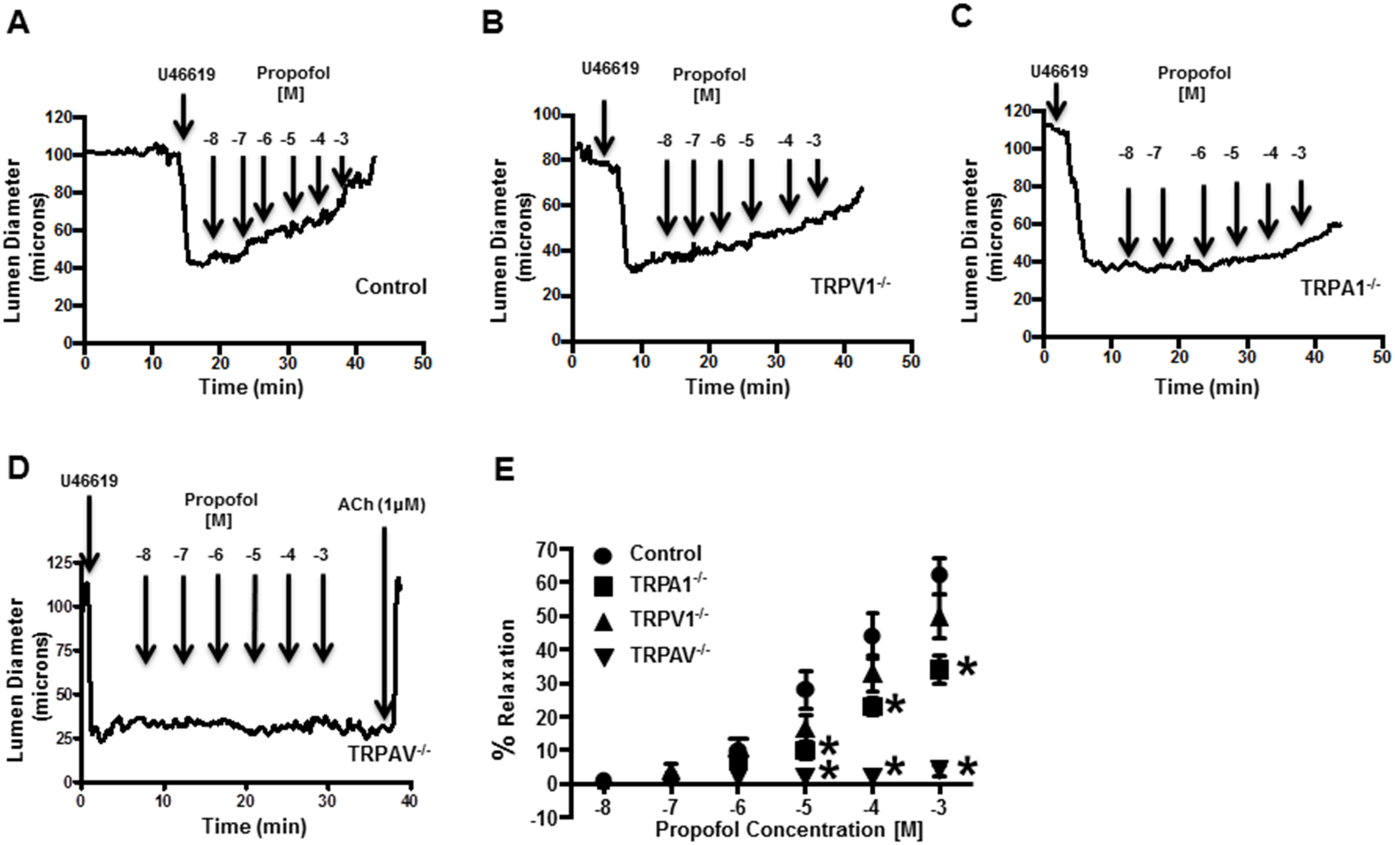
**A**: Representative trace depicting the effect of propofol (10^−7^ and 10^-3^M) on luminal diameter in an isolated coronary microvessel obtained from a control mouse pre-constricted with the thromboxane mimetic U46619 (1 μM). **B-D**: Same as panel A except in a coronary microvessel obtained from a TRPV1^-/-^ mouse. a TRPA1^-/-^ mouse or a TRPAV^-/-^ mouse, respectively. **E**: Summarized data depicting the effect of propofol on luminal diameter in isolated coronary microvessels obtained from control (*n* = 12), TRPV1^-/-^ (*n* = 12) TRPA1^-/-^ (*n* = 15) and TRPAV^-/-^ (*n* = 12). Data are expressed as % relaxation ± SEM. *P< 0.05 vs. control.

To evaluate the contribution of TRPA1 and TRPV1 to propofol-mediated vasoreactivity, coronary microvessels were isolated from hearts of all 4 groups of mice. Following pre-constriction of the vessels with the thromboxane mimetic, U46619 (1 uM), administration of propofol caused a dose-dependent relaxation of microvessels obtained from control mice (Fig 1A). The propofol-induced vasorelaxation was not affected in TRPV1^-/-^, markedly attenuated in TRPA1^-/-^ and abolished in microvessels obtained from TRPAV^-/-^ mice (Fig 1B–1D). The Intralipid vehicle had no effect on luminal diameter at any concentration tested. Summarized data depicting the effect of propofol on vasorelaxation in control, TRPA1^-/-^, TRPV1^-/-^ and TRPAV^-/-^ mice are depicted in Fig 1E. We also perfomed additional control experiments to confirm the specificity of propofol for TRPA1 channels using a TRPV1 antagonist in microvessels obtained from TRPA1^-/-^ mice. We found TRPV1 inhibition with SB366791 (10 μM) significantly blunted the propofol-mediated (1 mM) vasorelaxation (36 ± 4.3% vs 19 ± 3.8% relaxation; n = 4). However, TRPA1 inhibition with HC-030031 (10 μM) in microvessels obtained from TRPV1^-/-^ mice virtually abolished the propofol-mediated vasorelaxation (54 ± 5.7% vs 5 ± 4.2% relaxation; n = 4)

### Contribution of eNOS and/or BK_Ca_ channels to propofol-induced vasoreactivity

**Fig 2:**
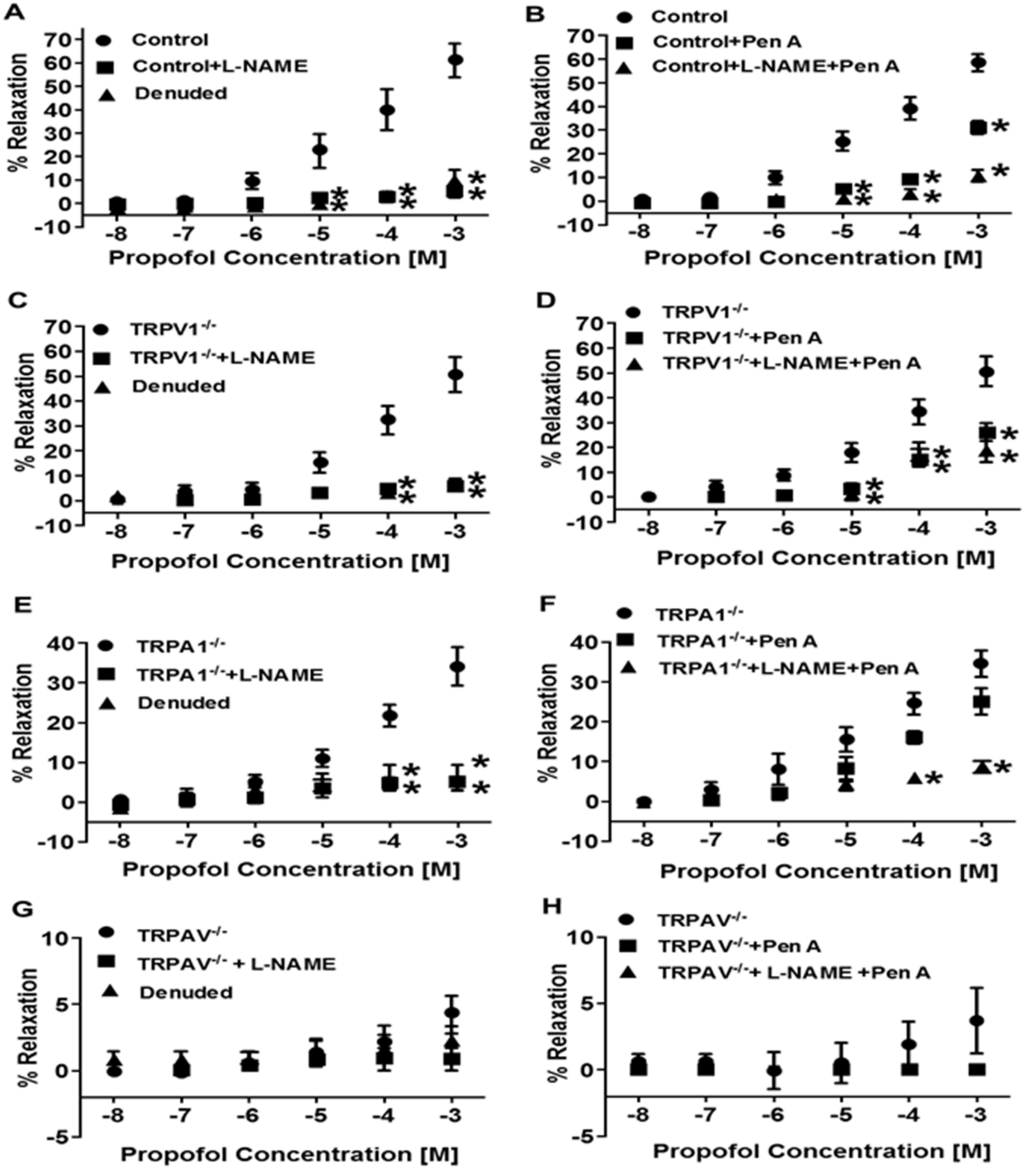
**A, C, E and G**: Summarized data depicting the effect of L-NAME (100 μM) or endothelium removal (denuded) on propofol-induced (does-dependent) changes in luminal diameter in coronary microvessels obtained from control, TRPV1^-/-^, TRPA1^-/-^ and TRPAV^-/-^ mice, respectively (*n* = 12). **B, D, F and H**: Summarized data depicting the effect of Pen A (50 μM) alone and in combination with L-NAME on propofol-induced changes in luminal diameter in coronary microvessels obtained from control, TRPV1^-/-^, TRPA1^-/-^ and TRPAV^-/-^ mice, respectively (*n* = 12). Data are expressed as % relaxation ± SEM. *P< 0.05 vs. control.

The extent to which NO and BK_Ca_ channels are involved in mediating the propofol-induced relaxation were next examined in isolated coronary microvessels. L-NAME and Pen A were utilized, alone and in combination, to inhibit eNOS and BK_Ca_, respectively. In addition, we also performed separate experiments in endothelium-denuded microvessels. In microvessels obtained from control mice, L-NAME virtually abolished propofol-induced vasorelaxation (Fig 2A). Similarly, Fig 2A also demonstrates that propofol-induced vasorelaxation is also abolished in endothelium denuded microvessels. Moreover, Pen A alone also caused a marked reduction in propofol-induced vasorelaxation in vessels obtained from control mice while combination of L-NAME and Pen A virtually abolish the propofol effect (Fig 2B). Similar results were obtained in microvessels obtained from TRPV1^-/-^ mice (Fig 2C and 2D). In microvessels obtained from TRPA1^-/-^ mice, L-NAME or endothelial denuding again abolished the already blunted propofol-induced vasorelaxation (Fig 2E) and while Pen A had little effect in microvessels obtained from TRPA1^-/-^ mice, the combination of L-NAME and Pen A abolished propofol-induced vasorelaxation (Fig 2F). In microvessels obtained from TRPAV^-/-^ mice, propofol-induced vasorelaxation was completely abolished and therefore addition of L-NAME or Pen A (alone or in combination) had no additional effect (Fig 2G and 2H).

### Assessment of TRP channel expression in MCAECs

**Fig 3:**
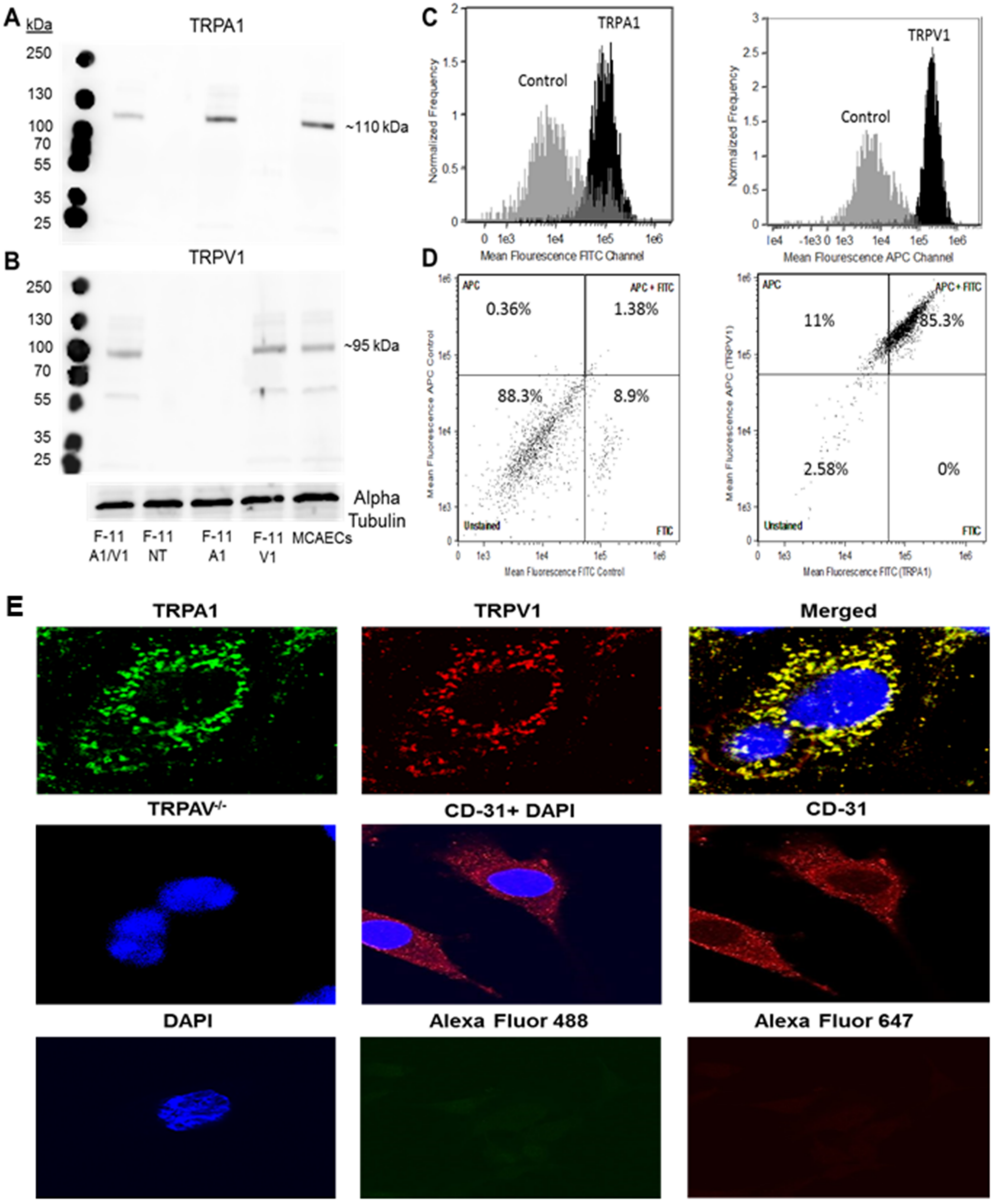
**A and B**: Representative immunoblot images depicting the expression of TRPA1 and TRPV1 in TRPA1- TRPV1 co-transfected F-11 cells (F-11 V1/A1), MCAECs and non-transfected F-11 cells (F-11 NT). Alpha Tubulin was used as loading control. **C**: Fluorescent activated cell-sorting (FACS) analysis of MCAECs exposed to isotype control sera (grey traces), specific-TRPA1 antisera (black traces) or specific-TRPV1 antisera (black traces). **D**: FACS analysis of MCAECs co-stained with both TRPA1 and TRPV1 antisera depicting % of cell population co-expressing TRPA1 and TRPV1. Cells co-stained with rabbit and mouse isotype control antisera were used as a negative control. **E**: Immunocytochemical localization of TRPA1 and TRPV1 in MCAECs. Green represents TRPA1 localization, red represents TRPV1 localization and yellow represents (merged) the co-localization of TRPA1 and TRPV1 (top row). There was no immunoreactivity for TRPA1 or TRPV1 in aortic endothelial cells obtained from TRPAV^-/-^ mice and CD- 31 staining with and without DAPI was used as an endothelial cell marker (middle row). MCAECs stained with DAPI (blue), Alexa Fluor 488 or Alexa Fluor 647 only were used as negative controls (bottom row). *n* = 5 different cell lysates or plates of cells.

Endothelial TRPA1 and TRPV1 channel expression in MCAECs were evaluated and confirmed using immunoblot analysis, as shown in Fig 3A and 3B respectively. Immunoblot analysis detected a specific band at ~110 (TRPA1) and ~95 (TRPV1) kDa respectively, indicating an endogenous expression of both channels in MCAECs. TRPA1 and TRPV1 co-transfected F-11 cells, TRPA1 only transfected, TRPV1 only transfected and non-transfected F-11 cells were used as positive and negative controls respectively.

To further consolidate our findings and validate the specificity of the TRPA1 and TRPV1 antisera, FACS analysis was performed to determine the cellular expression patters of TRPA1 and TRPV1. We observed that MCAECs stained with either TRPA1 or TRPV1 antisera resulted in an increase in mean fluorescence value compared to the cells stained with isotype-control antisera (Fig 3C). Moreover MCAECs co-stained with rabbit-TRPA1 conjugated with FITC and mouse-TRPV1 conjugated with allophycocyanin (APC) revealed that approximately 85.3% cells stained for both TRPA1 and TRPV1. Nearly 11% of the cells stained for TRPV1 only, whereas no cells stained for TRPA1 only and ~2.58% cells were unstained (Fig 3D). Cells co-stained with rabbit and mouse isotype control antisera conjugated with FITC and APC respectively were used as negative control.

Immunocytochemical localization of TRPA1 and TRPV1 with TRPA1 antisera conjugated with Alexa Fluor 488 secondary antibody and TRPV1 antisera conjugated with Alexa Fluor 647 respectively are depicted in Fig 3E **(top row)**. Concentrated localization of both TRPA1 and TRPV1 was detected along the perimeter of MCAECs. There were no immunodetectable TRPA1 or TRPV1 fluorescent signals observed in aortic endothelial cells obtained from TRPAV^-/-^ mice further supporting the specificity of the generated antisera (middle row). The endothelial cell marker CD-31 with and without DAPI (middle row), was used to confirm the specificity of the cells (middle row) and MCAECs stained with DAPI (blue), Alexa Fluor 488 or Alexa Fluor 647 only were used as negative controls (bottom row).

### Characterizing TRPA1 and TRPV1 ion channel properties in MCAECs

**Fig 4:**
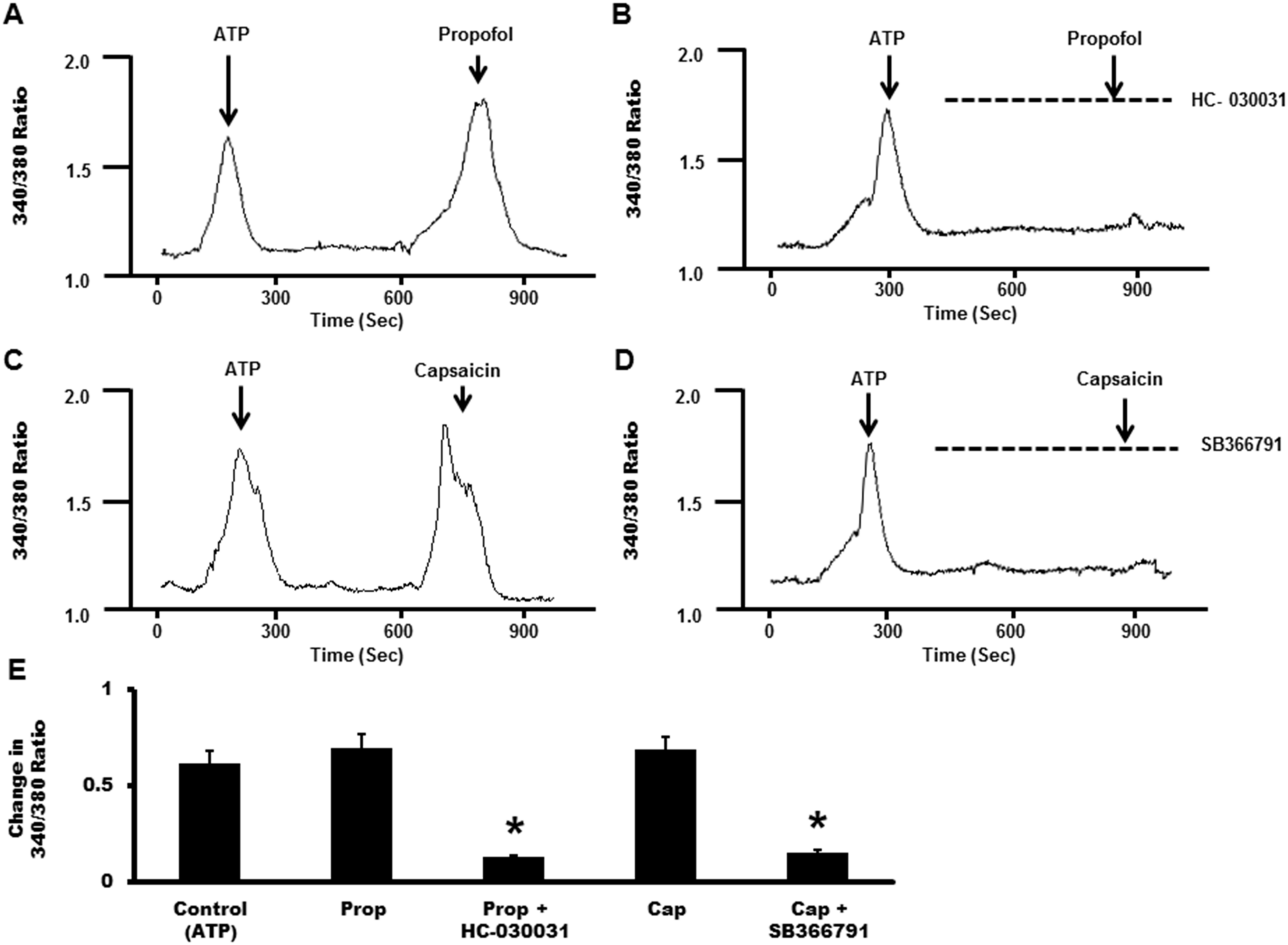
Representative calcium imaging traces depicting the effect of single application of (**A**) propofol (prop; 10 μM), a TRPA1 agonist; (**B**) propofol in the presence of HC-030031 (1 μM), a TRPA1 antagonist; (**C**) capsaicin (cap; 100 nM), a TRPV1 agonist and (**D**) capsaicin in the presence of SB366791 (5 μM), a TRPV1 antagonist. ATP (10 μM) was used as an internal control representing the expected maximal response. **E**: Summarized data for Fig 4A–4D. Data are expressed as changes in the raw 340/380 ratio values ± SEM in response to the intervention. *P< 0.05 vs. propofol or capsaicin treated cells. *n* = 6 separate cover slips of MCAECs were used.

MCAECs containing functional TRPA1 and TRPV1 receptors were identified with a single application of propofol (10 μM) and capsaicin (100 nM). Application of both propofol and capsaicin resulted in a transient increase in intracellular-calcium [Ca^2+^]_i_ levels (Fig 4A and 4C). Moreover, propofol and capsaicin-induced transient increases in [Ca^2+^]_i_ were abolished in the presence of HC-030031 (1 μM), a TRPA1 inhibitor and SB366791 (5 μM), a TRPV1 inhibitor, respectively (Fig 4B and 4D). Cell viability was assessed with a single application of ATP (10 μM) to ensure receptor-effector coupling is intact in cultured MCAECs. Summarized data depicting the effect of propofol and capsaicin alone as well as in the presence of HC-030031 or SB366791 are illustrated in Fig 4E. Summarized data are expressed as a percent of the mean change from baseline value, which was normalized to 100%.

### Effect of propofol-induced stimulation of TRPA1 on direct physical interaction between TRPA1-TRPV1 in MCAECs

**Fig 5:**
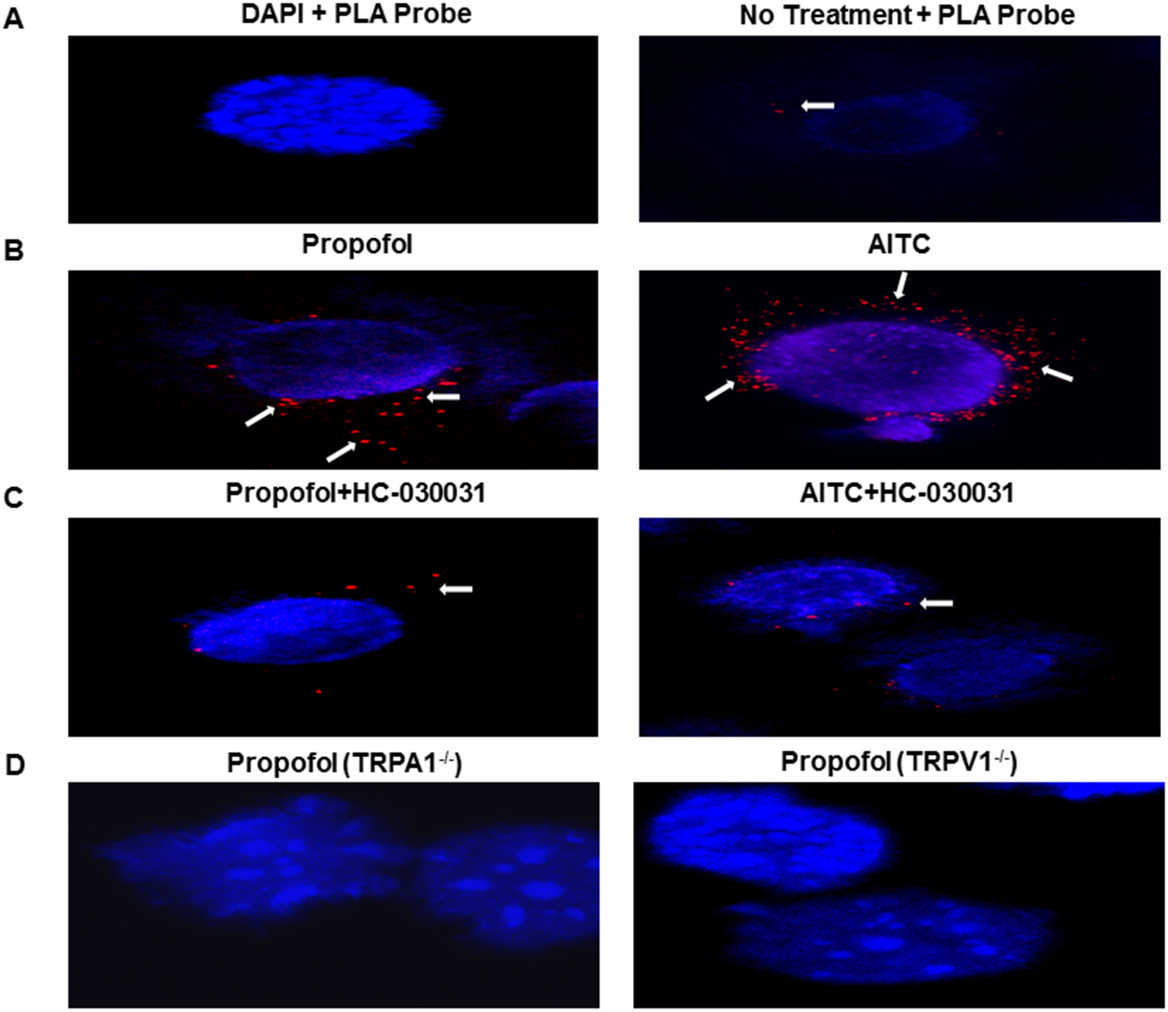
Detection of TRPA1-TRPV1 complex formation in MCAECs using proximity ligation assay (PLA). **A**: MCAECs stained with PLA probes only were used as negative controls. MCAECs stained with DAPI (blue), TRPA1 and TRPV1 antisera conjugated with PLA probes (red signal). **B**: Effect of propofol (10μM) or AITC (100μM) treatment on PLA signal in MCAECs. **C**: PLA of MCAECs pretreated with HC-030031 (0.5μM), then incubated with either propofol (10μM) or AITC (100μM). **D**: Lack of effect of propofol (10 μM) on aortic endothelial cells obtained from TRPA1^-/-^ and TRPV1^-/-^ mice. *n* = 4 coverslips of cells.

To examine the extent to which propofol-induced activation of TRPA1 leads to a physical interaction between the two transmembrane receptors in their native environment, we employed a PLA on cultured MCAECs using rabbit TRPA1 and mouse TRPV1 antisera. Compared to the non-treated cells which showed minimal PLA signals (Fig 5A), a robust signal was detected in cells treated with either propofol (10μM) or allyl isothiocyanate (AITC) (100μM; Fig 5B). Moreover, the effect of propofol or AITC was markedly attenuated in cells pretreated with HC-030031 (0.5μM), a TRPA1- specific inhibitor (Fig 5C). Furthermore, no signals were observed in mouse aortic endothelial cells obtained from TRPA1^-/-^ or TRPV1^-/-^ mice treated with propofol (10μM) (Fig 5D). Lack of PLA signals in DAPI and untreated plus PLA probe (Fig 5A) as well as TRPA1 and TRPV1 knockouts (Fig 5D) confirms the specificity of the assay.

## Discussion

Our laboratory recently demonstrated a novel role for TRPA1 and TRPV1 ion-channels in mediating propofol-induced depressor responses *in* vivo [1]. Moreover, recent studies have demonstrated a vasodilatory component following propofol administration in isolated coronary artery preparations [7, 9, 20] however the extent to which the endothelium is involved appears to be controversial [7, 20, 21] but a role for the activation of BK_Ca_ channels has been previously reported [20]. In the current study, we have extended our previous *in vivo* studies and examined the extent to which TRPA1 and/or TRPV1 are involved in mediating propofol-induced relaxation responses in isolated coronary arterioles *in vitro*. We report that propofol induces a dose-dependent relaxation of pre-constricted coronary microvessels obtained from control mice, maintained in vessels obtained from TRPV1^-/-^ mice, markedly attenuated in TRPA1^-/-^ mice and completely abolished in TRPAV^-/-^ mice. In TRPA1^-/-^ microvessels, the TRPV1 antagonist SB-366791 significantly blunted the propofol-induced vasorelaxation. However, in TRPV1^-/-^ microvessels, the TRPA1 antagonist HC-030031 virtually abolished the propofol-induced vasorelaxation. These data suggest that in the absence of TRPV1, the propofol-induced effects are mediated by TRPA1 whereas in the absence of TRPA1, the vasorelaxant effects of propofol are mediated by TRPV1. TRPV1 channels have been reported to mediate increases in coronary blood flow *in vivo*, and vasorelaxation of coronary microvessels *in vitro* via NO and BK_Ca_ channels [5, 22]. In addition, although previous reports clearly indicate propofol activates TRPA1 in sensory neurons, there are conflicting reports as to whether propofol activates TRPV1 [12–14]. However, functional cross regulation between TRPV1 and TRPA1 channels whereby TRPV1 modulates TRPA1-mediated responses in the regulation of nociceptive transmission has been demonstrated, primarily via direct molecular interactions between the two channels forming heterotetramers [23–25]. Modulation of TRPA1 by TRPV1 suggests that the TRPA1 density is heavily regulated by TRPV1, and TRPV1 directly affects physiological TRPA1 activity [26, 27]. Therefore, if TRPV1 serves as a regulatory subunit of a TRPA1-TRPV1 complex, then modification or deletion of TRPV1 presumably would alter the expression and activity or perhaps the sensitivity of TRPA1. This could also apply to ligands whereby the ligand could either facilitate or inhibit the activity of TRPA1. Although, many studies have implicated TRPV1 as a regulator of TRPA1 function, the current data suggest that TRPA1 may be a regulator of TRPV1 in the coronary vasculature. This is consistent with our previous findings in sensory neurons demonstrating a propofol-induced, TRPA1-mediated regulation of TRPV1 sensitivity to agonist activation via a PKCε-dependent pathway [11, 28]. Thus, crosstalk between the channels can occur in both directions and could be mediated either through a direct molecular interaction between the channels and/or via intracellular mediators.

We performed experiments in pre-constricted coronary microvessels to assess the degree to which NO and BK_Ca_ channels are involved in mediating the propofol-induced relaxation response. Our findings indicated that propofol-induced vasorelaxation is endothelium-dependent since denuded vessels from all four groups of mice fail to relax upon administration of propofol. Similarly, NOS inhibition completely blocked the vasorelaxant effect of propofol in all mice. In addition, propofol-induced vasorelaxation was virtually abolished by BK_Ca_ channel inhibition in vessels from control, TRPV1^-/-^ and TRPAV^-/-^ mice whereas the magnitude of inhibition was not as great as that observed in vessels obtained from TRPA1^-/-^ mice. Taken together, these data indicate a complex regulation of vascular tone by both TRPA1 and TRPV1 signaling pathways. The BK_Ca_ channel component of the propofol-induced relaxation effect appears to be mediated by TRPA1 because little additional effect of Pen A was observed in vessels obtained from TRPA1^-/-^ mice. Because NOS inhibition completely abolished propofol-induced vasorelaxation in both TRPA1^-/-^ and TRPV1^-/-^ mice, and the propofol-induced effect is abolished in TRPAV^-/-^ mice in the absence of NOS inhibition suggests that both TRPV1 and TRPA1 can regulate NOS activity which depends on whether only one channel or both channels are expressed. This could be explained if TRPA1 is acting as a negative regulator of TRPV1 as proposed above. In the absence of TRPV1, stimulation of TRPA1 by propofol results in eNOS activation and NO production to elicit vasorelaxation. In the absence of TRPA1, the negative regulation of TRPV1 is removed allowing for activation by propofol and/or an increase in the sensitivity of the channel to propofol resulting in TRPV1-dependent activation of NOS, NO production and a vasorelaxant effect. Alternatively, ATP-gated K+ channels and cyclooxygenase products may contribute to the propofol-mediated relaxation, suggesting a role for other signaling mediators in the response [29, 30]. Further studies are required to confirm the extent to which TRPA1 serves as a negative regulator of TRPV1 activation/sensitivity in the coronary vasculature.

The potential physiological and pathophysiological roles of TRPA1 [3, 31] and TRPV1 [22, 32, 33] in the vascular system have previously been reviewed. However, there is a lack of data demonstrating the functional expression and localization of either channel in coronary artery endothelial cells. Our functional data obtained from the knockout mice clearly predicts the presence of functional channels in the coronary microvasculature. We therefore performed immunoblot analysis for expression of TRPA1 and TRPV1 in MCAEC’s, FACS analysis to assess the population of MCAEC’s expressing and/or co-expressing TRPA1 and TRPV1 and immunocytochemistry to identify the intracellular localization of the channels. Our immunoblot analysis in indicates both channels are expressed in MCAECs. Moreover, FACS analysis revealed that there are two subpopulations of MCAECs expressing TRPV1 only (11%), TRPV1 and TRPA1 (85%) as well as one subpopulation that do not express either channel (2.5%). These data indicate that the majority of MCAECs co-express both TRPA1 and TRPV1, which is likely responsible for the functional effects observed of propofol-induced vasorelaxation in coronary arterioles. As predicted, immunocytochemical analysis of channel expression indicated both channels were immunolocalized at the periphery of MCAECs. We next assessed the extent to which the channels in MCAECs are functional and allow for the passage of Ca^2+^ into the cell following receptor activation in the presence and absence of specific receptor antagonists.

Regulation of TRPA1 and TRPV1 ion-channel properties and modulation of these channels in the coronary microcirculation could be of tremendous importance for better understanding coronary blood flow regulation both in the physiological and pathophysiological settings as previously reviewed [3, 22, 31, 34, 35]. It is well established in sensory neurons [11, 13] and in other tissue types [36] that receptor activation with capsaicin (specific TRPV1 agonist) or allyl isothiocyanate (TRPA1 agonist) results in channel activation and a transient influx of Ca^2+^ leading to an increase in intracellular free Ca^2+^ concentration [Ca^2+^]_i_. Although there is no evidence of propofol-induced TRPV1-mediated calcium-influx, we previously demonstrated that propofol induces a transient increase in [Ca^2+^]_i_ via a TRPA1-dependent pathway in sensory neurons [11]. To confirm the functional integrity of these channels in MCAECs, we performed experiments to assess the change in [Ca^2+^]_i_ in response to channel specific agonists, in presence or absence of TRPA1 or TRPV1 antagonists. Our findings indicate that MCAECs respond to capsaicin (TRPV1 channel agonist) and propofol (TRPA1 channel agonist) with a transient increase in [Ca^2+^]_i_. Moreover, the capsaicin and propofol-induced transient rise in [Ca^2+^]_i_ was suppressed in the presence of SB366791 (a specific TRPV1 antagonist) and HC-030031 (a TRPA1 antagonist), respectively. These data demonstrate functionally active endothelial TRPA1 and TRPV1 channels and is consistent with the channels acting as non-selective cation channels as previously described by our laboratory and others [11]. We next assessed the extent to which heterodimerization between TRPA1 and TRPV1 occurs following TRPA1 activation.

Modulation of TRPA1 by TRPV1 via a direct interaction between the channels has been reported previously [25, 37, 38]. Heterodimer formation between TRPA1 and TRPV1 channels results in dramatic effects on channel biophysical properties, pharmacology, signaling, regulation, and ultimately function [23, 24, 39]. A TRPV1- TRPA1 interaction is thought to be predominantly calcium-dependent, resulting in heteromeric channel formation [23, 27, 37, 38]. Moreover, this complex displays a threshold and temperature coefficient similar to TRPV1. Although evidence to support activation of TRPV1 leading to a direct molecular interaction between TRPV1 and TRPA1 in heterologous expression systems and sensory neurons [27, 40], there is no evidence that TRPA1 activation leads to a direct physical interaction between TRPA1 and TRPV1 particularly in the vascular system. As noted above, expressions of TRPA1 and TRPV1 in MCAECs results in co-localization near the periphery of cell allowing for the potential for a direct molecular interaction between the two channels. Our PLA data uniquely demonstrate that both propofol and AITC-induced activation of TRPA1 triggers a direct molecular-interaction between TRPA1 and TRPV1, which is markedly reduced in the presence of the TRPA1 inhibitor, HC-030031. Collectively, these data substantiate our current findings and likely is a key factor related to both TRPA1 and TRPV1 contributing to propofol-mediated antagonism of the U46619-mediated vasoconstriction observed in the microvascular reactivity studies. Therefore it appears that directional cross-talk from TRPA1 to TRPV1 may be of great importance in modulating coronary microvascular regulation and suggests that either channel is capable of potentially regulating/modulating the other.

## Summary and conclusion

Our study indicates that the propofol-mediated relaxation in isolated coronary microvessels involves endothelium TRPA1-dependent signaling whereas endothelial TRPV1-mediated signaling appears to be absent. Moreover, inhibition of eNOS virtually abolishes the effect of propofol consistent with the dependence of an intact endothelium in order to observe the propofol effect. Furthermore, BK_Ca_ channel activation contributes to the TRPA1-dependent signaling cascade. Collectively, these data suggest TRP channel stimulation leads to NO production and subsequent BK_Ca_ channel activation. Alternatively, ATP-gated K+ channels and cyclooxygenase products may contribute to the propofol-mediated relaxation, suggesting a role for other signaling mediators in the response [29, 30]. Similarly, in the framework of former studies regarding TRPV1-mediated relaxation, previous studies from our lab suggests a distinct role for TRPV1 to evoke coronary vasorelaxation [41, 42]. However, it should be noted that capsaicin-mediated vasorelaxation has also been shown to display TRPV1-independent mechanisms [43] whereas others have demonstrated submicromolar capsaicin evokes coronary vasoconstriction [44]. In conclusion, the current study clearly demonstrates a role for TRP ion channels in propofol-mediated antagonism of U46619-induced vasoconstriction in isolated murine coronary arteries.
